# Artificial intelligence-assisted esophagogastroduodenoscopy improves procedure quality for endoscopists in early stages of training

**DOI:** 10.1055/a-2547-6645

**Published:** 2025-04-15

**Authors:** Shannon Melissa Chan, Daniel Chan, Hon Chi Yip, Markus Wolfgang Scheppach, Ray Lam, Stephen KK Ng, Enders Kwok Wai Ng, Philip W Chiu

**Affiliations:** 1 26451 Department of Surgery, The Chinese University of Hong Kong, Hong Kong, Hong Kong; 2 4333 Surgery, UNSW St George & Sutherland, Kogarah, Australia; 3 531257 Internal Medicine III - Gastroenterology, University of Augsburg Faculty of Medicine, Augsburg, Germany

**Keywords:** Quality and logistical aspects, Training, Image and data processing, documentatiton, Quality management

## Abstract

**Background and study aims:**

Completeness of esophagagogastroduodenoscopy (EGD) varies among endoscopists, leading to a high miss rate for gastric neoplasms. This study aimed to determine the effect of the Cerebro real-time artificial intelligence (AI) system on completeness of EGD for endoscopists in early stages of training.

**Patients and methods:**

The AI system was built with CNN and Motion Adaptive Temporal Feature Aggregation (MA-TFA). A prospective sequential cohort study was conducted. Endoscopists were taught about the standardized EGD protocol to examine 27 sites. Then, each subject performed diagnostic EGDs per protocol (control arm). After completion of the required sample size, subjects performed diagnostic EGDs with assistance of the AI (study arm). The primary outcome was the rate of completeness of EGD. Secondary outcomes included overall inspection time, individual site inspection time, completeness of photodocumentation, and rate of positive pathologies.

**Results:**

A total of 466 EGDs were performed with 233 in each group. Use of AI significantly improved completeness of EGD [mean (SD) (92.6% (6.2%) vs 71.2% (16.8%)]; *P* <0.001 (95% confidence interval 19.2%–23.8%, SD 0.012). There was no difference in overall mean (SD) inspection time [765.5 (338.4) seconds vs 740.4 (266.2); *P* =0.374]. Mean (SD) number of photos for photo-documentation significantly increased in the AI group [26.9 (0.4) vs 10.3 (4.4); *P* <0.001]. There was no difference in detection rates for pathologies in the two groups [8/233 (3.43%) vs 5/233 (2.16%), *P* =0.399].

**Conclusions:**

Completeness of EGD examination and photodocumentation by endoscopists in early stages of are improved by the AI-assisted software Cerebro.

## Introduction

Esophagogastroduodenoscopy (EGD) is the standard luminal diagnostic tool for detection of esophageal, gastric, and duodenal disorders. However, Menon et al. showed that 11.4% of upper gastrointestinal (UGI) cancers are not detected by EGD in the 3 years before diagnosis 1 . The Japanese working group for quality assurance of endoscopic screening for gastric cancer recommends taking 30 to 40 images for a complete examination of the stomach, which should then be reviewed by experts for content and image quality 2 . In its quality improvement initiative, the European Society of Gastrointestinal Endoscopy (ESGE) and the American Gastroenterological Association clinical practice update list accurate photo-documentation of anatomic landmarks as well as an examination time of more than 7 minutes as relevant performance measures for UGI endoscopy 3 4 . An examination time above this cutoff (7 mins) was associated with a twofold increase in detection of high-risk gastric lesions such as atrophic gastritis and intestinal metaplasia, and a threefold increase in detection of dysplastic lesions and gastric cancers in a Singaporean study 5 . In another ESGE position statement about artificial intelligence (AI) in gastrointestinal endoscopy in 2022, it was agreed that rate of complete inspection of the UGI mucosa and percentage of reports with adequate photo-documentation should be part of the performance measures 6 . In Japan, a protocol for examination of the stomach was proposed by Yao 7 . Herein, a complete EGD should include 22 standardized images of the stomach 7 (gastric mucosa are required in forward view (4 from the antrum, 4 from the fundus/cardia, 4 from the body) and retroflexed view (4 from the fundus/cardia, 3 from the body, 3 from the angular incisura)) and images from the esophagus and duodenum 3 . Due to their complexity, these measures of procedure time and inspection quality seem hard to follow rigorously in clinical practice, especially for trainees.

In recent years, AI algorithms have been successfully implemented in gastrointestinal endoscopy 8 9 10 11 12 . However, most research endeavors focus on detection, delineation, and characterization of gastrointestinal lesions, such as esophageal 12 13 and gastric 9 cancers and their precursors. Wu et al. developed an AI system to improve completeness of endoscopic inspection during EGD with implementation of a real-time AI system 14 ; in this randomized controlled trial, the rate of blind spots of experienced endoscopists (2000–5000 EGDs) was reduced from 22.46% to 5.85%. These results show the potential of AI algorithms to improve endoscopic inspection quality during EGD by experienced endoscopists.

Therefore, we developed an AI algorithm named Cerebro (Endovision Limited, Hong Kong) which supports endoscopists in completing a standardized protocol of complete EGD examination and high-quality image recording ( Video 1 ). Our focus for clinical evaluation of this algorithm was on inexperienced endoscopists during training, which requires supervision (< 250 EGDs) 15 , because this group may benefit most from continuous AI support. The aim of this study was to evaluate whether use of this AI algorithm can improve completeness of EGD by unexperienced endoscopists during training.

## Methods

Training and validation of the AI system was done on the following dataset using annotated still images. These videos and images were collected in the Prince of Wales Hospital, Hong Kong. A total of 1345 videos and 1,593,856 frames were used; 791 video and 532,930 frames for training, and 554 videos with 1,060,926 frames for validation.

A complete EGD is defined as having examined the 22 sites of the stomach from the protocol by Yao 7 together with five extra sites 3 , including the esophagus, z-line, fornix of the fundus, duodenal bulb, and second part of duodenum pars descendens, i.e. 27 sites total. Using the above training dataset, a scene (spatial) perception model was trained using EfficientNet-B7 core architecture. In addition, smaller classifiers and single shot detector (SSD) models were trained using YoloV5 architecture to support the scene perception module. Temporal perception was built using a graph-based time-series approach called Motion Adaptive Temporal Feature Aggregation (MA-TFA).

Detailed information about all the models and datasets can be found in Supplementary Material 1.

**Fig. 1:**
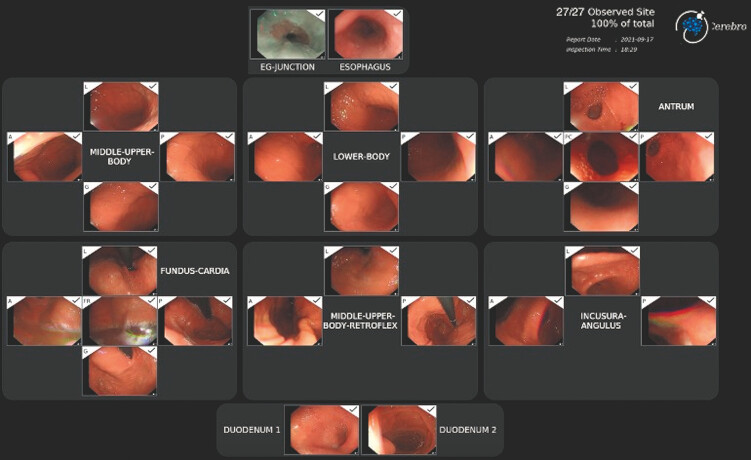
Final photo-documentation of Cerebro.

The resulting application generated the following outputs during EGD in real time:

1. Inspection completeness of all the aforementioned sites is detected and depicted in real time. The endoscopist receives feedback about completeness of each individual site in real time.
2. A timer measures examination time between scope insertion into the mouth and extubation from the mouth and provides individual inspection times for each site. An inspection time of minimum 2 seconds is required at each individual site before that site is defined as “complete”.
3. When the endoscopic view is inadequate, necessity for gas insufflation or mucosal irrigation is displayed.
4. Storage of the most representative image from each site is performed by the software and presented at the end of the examination in the form of an automatic report ( Fig. 1 )

For validation of the software, 100 random full-length EGD videos were separately collected. These were fed into the algorithm. The videos together with the software output were screened by three expert endoscopists (EGD experience > 8 years, > 500 cases per year) for correct identification of the anatomic sites. For each site, a binary decision of true or false identification was made. Any discrepancy in the decision was resolved by consensus among the three experts. For each patient, an overall sensitivity, specificity, and accuracy were derived. The experts also checked accuracy of the photos recorded for the final photodocumentation.

### Clinical evaluation

For clinical evaluation of the algorithm, a prospective sequential cohort study was conducted from May 2021 to October 2021. The aim of the study was to compare completeness of EGD with AI (control arm) and without AI (study arm). Eight endoscopy trainees with an experience of 50 to 100 endoscopies were recruited for this study. First, these subjects were taught about the mentioned 27 sites as a standardized EGD protocol as part of inhouse training. They were taught two didactic lectures on basic endoscopic techniques and the quality indicators of EGD. These included Yao et al.’s proposal of the 22 sites examination in the stomach 7 and the five additional sites in the esophagus and duodenum 3 which were proposed in this study. Then, each subject performed diagnostic EGDs per protocol (control arm). Subjects were then briefed on the function of the AI-algorithm and given 2 weeks to get accustomed to the system. Then, enrolled endoscopists performed diagnostic EGDs with assistance of the AI (study arm). All examinations were recorded in full on video. Ethics approval was obtained from the Joint CUHK-NTEC clinical and research ethics committee. (ClinicalTrials.gov ID: NCT04883567) *.* The study was conducted in compliance with Declaration of Helsinki and ICH-GCP guidelines.

For the control arm, endoscopists performed EGD according to what they have been taught about inspecting the 27 sites. For the study arm, the endoscopists had real-time feedback and could modify their procedure accordingly to achieve completeness. All videos (including the non-AI and AI arm) were independently assessed by three expert endoscopists. The assessors were blinded to video groupings. Differences between assessor results were resolved by mutual consensus. All EGD videos (non-AI and AI) were also assessed retrospectively by the AI system for total procedure time and individual stie inspection time. The amount of photo-documentation per procedure was assessed retrospectively in the EGD report.

### Subject recruitment and procedure

All patients aged 18 years or older undergoing diagnostic EGD for evaluation of their symptoms in the Prince of Wales Hospital were included in the study. Exclusion criteria included patients for whom a full endoscopic examination was not required, patient condition requiring therapeutic endoscopy, early termination of endoscopy due to patient intolerance, presence of a large amount of food residue, presence of mechanical obstruction or for reasons of safety, patients with altered anatomy, pregnant patients, patients who refused to participate in the study and patients who were unfit to give consent.

Consents were signed before the procedure. Patients were given oral N-acetylcysteine 30 minutes before the procedure. Procedures were performed with the patient in the left lateral position with mouthguard, using a 10.2-mm flexible video gastrointestinal scope (EVIS Lucera Elite Gastrointestinal Videoscope, GIF-H290) (Olympus Medical Corp, Olympus Hong Kong and China Limited.). Air insufflation with high flow setting was used in all patients. All patients were given eight puffs of 10% topical xylocaine before the procedure unless contraindicated. Conscious sedation was given at endoscopist discretion as per usual practice in our center. All EGD videos were recorded.

### Outcomes

The primary outcome was the rate of completeness of EGD. The rate of completeness of EGD was defined as the number of covered sites divided by the total number of sites (27). Secondary outcomes included overall inspection time, individual site inspection time, completeness of photodocumentation, and rate of positive pathology. Positive pathology was defined as malignancy including esophageal, gastric, or duodenal cancers and subepithelial tumors.

### Sample size estimation

Sample size estimation was performed based on trial data from Wu et al. 14 , where the blind spot rate was 22.46% and 5.86% for examination without and with AI support respectively. From June 1, 2019 to July 1, 2019, 50 outpatient OGD procedures were randomly selected and reviewed by one expert endoscopist. Forty-two of the procedures (84%) were performed by endoscopists-in-training. The average blind spot rate was 24% with the most common blind spot sites being the fundus/cardia lesser curve side and the middle upper body lesser curve side. To demonstrate an estimated 10% improvement in EGD completeness by AI support with a power of 0.8 and a two-sided significance level of 5%, a sample size of 416 patients was calculated. Assuming an exclusion rate of 10%, it was planned to include 460 patients.

### Statistical analysis

For the statistical analysis, completeness of EGD and number of photos for photodocumentation were assessed using Poisson regression model because the two outcomes are rate (i.e., completeness of EGD) and counts (i.e., number of photos). Multivariable regression analyses with Poisson regression models were used to investigate the effect of AI-assisted OGD on these two outcomes, adjusted for age, gender, and the endoscopist who conducted the OGD as covariate.

All tests of significance were two-tailed and *P* < 0.05 was considered statistically significant. Statistical analyses were performed using SPSS 24.0 statistical software (SPSS, Chicago, Illinois, United States).

## Results

### Validation cohort

**Fig. 2:**
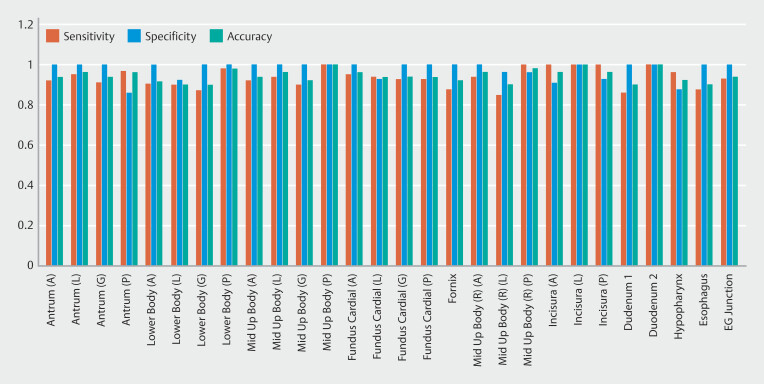
Sensitivity, specificity, and accuracy of Cerebro in validation study of 100 EGD videos.

Validation of 100 EGD procedures was performed and reviewed by three expert endoscopist. Fig. 2 shows sensitivity, specificity, and accuracy of these 100 validation videos. Average sensitivity, specificity, and accuracy of the 27 sites were 0.94, 0.98, and 0.94, respectively. Time for scope insertion and withdrawal was validated. Cerebro was able to predict the start time in 100% of the cases and 99% of the cases for withdrawal time.

### Prospective sequential cohort study

**Fig. 3:**
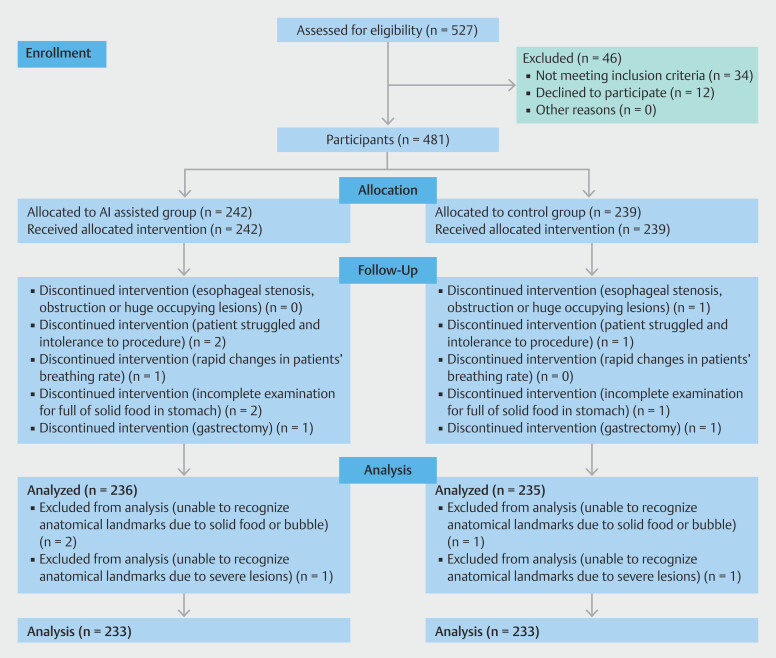
Study flow chart.

From May 2021 to October 2021, 466 procedures were performed and analyzed. Table 1 shows the basic demographic data. Mean (SD) age was 63.6 years (14.0) in the AI-assisted group and 64.1 years (15.8) in the control group. Of the patients, 54.1% were female in the AI-assisted group whereas 46.7% were female in the control group. The proportion of outpatient procedures in the AI group was 0.59 whereas that in the control group was 0.52. The percentage of patients who were sedated in the two groups was also similar. Indications for EGD are shown in Supplementary Material 2. There were five endoscopy trainees from surgery and three from gastroenterology. Fig. 3 shows the study flowchart. Of the participants, 481 were eligible with consents signed. However, 15 procedures were excluded due to stricture, incomplete procedure due to patient clinical condition, and also solid food residue in the stomach.

### Primary and secondary outcomes

Use of AI significantly improved completeness of EGD in the AI group as compared with control group (92.6 (6.2) % vs 71.2 (16.8) %; *P* < 0.001 (95% confidence interval [CI] 19.2%-23.8%] SE 0.012) ( Table 1 ). There was no difference in overall inspection time (765.5 ± 338.4 seconds vs 740.4 ± 266.2; 95%CI -30.3–80.5, SE 28.2; *P* = 0.374) ( Table 2 ). However, in the AI group, significantly more time was spent on inspecting the lower body (greater curve, lesser curve, anterior and posterior), mid-upper body forward view (greater curve, lesser curve, anterior and posterior), fundus (greater curve, lesser curve, anterior and posterior), middle-upper body retrospect view (lesser curve, anterior and posterior), and angular incisura (posterior). (Detailed timing for each location for both groups is included in Supplementary material 2). The number of photos for photo-documentation also significantly increased in the AI group (26.9 ± 0.4 vs 10.3 ± 4.4; 95% CI 16.0–17.2; *P* < 0.001; SE 0.20) ( Table 2 ). However, there was no difference in detection rates for pathologies in either group (8/233 [3.43%] vs 5/233 [2.16%], *P* = 0.399). Supplementary Table shows positive pathologies included in the two groups.

**Table:** **Table 1** Baseline demographic data in both groups.

	AI-assisted group n = 233	Control group n = 233	P value
M:F	107:126	109:124	0.853
Age (years)	63.6 (14.0)	64.1 (15.8)	0.733
BW (kg) mean (SD)	60.2 (12.4)	59.1 (10.9)	0.306
BH (meter) kg mean (SD)	160.3 (8.3)	160.1 (10.1)	0.795
BMI (kg/sq. meter) mean (SD)	23.3 (3.9)	23.2 (5.8)	0.778
Ethnicity			1.000
Chinese, no. of patient (%)	231 (99.1)	232 (99.6)	
Filipino, no. of patient (%)	2 (0.9)	1 (0.4)	
Sedation during EGD (No. of patients (%))	100 (42.9)	99 (42.5)	0.925
Proportion of outpatient procedures	137/233 (59%)	121/233 (52%)	0.759
AI, artificial intelligence; BMI, body mass index; BH, body height; BW, bodyweight; EGD, Esophagogastroduodenoscopy; SD, standard deviation.			

**Table:** **Table 2** Primary and secondary outcomes.

Outcome	AI group	Control group	Difference (95% CI)	SE	*P* value
Completeness of EGD Mean (SD)	**92.6%** (6.2%)	**71.2%** (16.8%)	**21.5%** (19.2%–23.8%)	1.2%	**< 0.001**
Overall inspection time (seconds) Mean (SD)	765.5 (338.4)	740.4 (266.2)	25.1 (–30.3–80.5)	28.2	0.374
No. of photos for photodocumentation	**26.9** (0.4)	**10.3** (4.4)	16.6 (16.00–17.2)	0.29	**< 0.001**
AI, artificial intelligence; CI, confidence interval; EGD, esophagogastroduodenoscopy; SD, standard deviation; SE, standard error.					

When considering the eight endoscopists separately, use of AI also improved completeness of EGD individually ( Table 3 ). The most commonly missed sites were the fundus-cardia region and lesser curve of upper and mid body ( Table 4 ).

**Table:** **Table 3** Effect of AI on each individual endoscopist regarding EGD completeness.

			Completeness of EGD mean (S.D.)	Completeness of EGD mean (S.D.)			
Endoscopist	No. of EGDs performed	AI group: control group (no. of patients)	AI group	Control group	Difference (95% CI)	SE	P value
1	50	25:25	95.4% (6.4%)	64.9 (14.2%)	30.5% (24.2%–36.9%)	3.1%	<0.001
2	50	30:30	90.6% (7.9%)	64.9% (16.2%)	25.7% (19.0%–32.3%)	3.3%	<0.001
3	100	30:30	95.3% (4.3%)	75.4% (17.5%)	19.9% (13.2%–26.5%)	3.3%	<0.001
4	100	30:30	93.1% (5.1%)	79.3% (15.9%)	13.8% (7.6%–20.0%)	3.0%	<0.001
5	100	30:30	91.7% (5.3%)	79.0% (13.3%)	12.7% (7.5%–18.0%)	2.6%	<0.001
6	50	30:30	89.8% (7.5%)	63.8% (21.1%)	25.9% (17.6%–34.2%)	4.1%	<0.001
7	50	30:30	93.7% (5.4%)	68.0% (13.8%)	25.7% (20.2%–31.2%)	2.7%	<0.001
8	50	28:28	91.9% (5.3%)	73.0% (12.7%)	18.9% (13.6%–24.2%)	2.6%	<0.001
AI, artificial intelligence; CI, confidence interval; EGD, esophagogastroduodenoscopy; SD, standard deviation; SE, standard error.							

**Table:** **Table 4** The inspection time patients in each site between AI assisted OGD and control group.

Inspection time	AI assisted (n=233) Mean (S.D.) time in seconds(sd)	Control (n=233) Mean (S.D.) time in seconds	P value
Overall	776.7 (341.8)	745.4 (267.9)	0.272
Oesophagus	22.1 (26.3)	20.8 (17.8)	0.536
Squamocolumnar junction	17.5 (23.4)	16.3 (19.1)	0.520
Antrum (G)	39.2 (27.8)	35.1 (23.1)	0.079
Antrum (P)	29.4 (21.5)	25.8 (20.3)	0.065
Antrum (A)	27.1 (24.5)	22.8 (22.6)	0.051
Antrum (L)	31.4 (25.5)	25.5 (23.8)	0.011
Duodenal bulb	14.7 (15.2)	13.7 (12.1)	0.417
Duodenal descending	31.8 (34.4)	35.9 (33.8)	0.196
**Lower body (G)**	**13.1 (10.0)**	**7.9 (9.1)**	***<0.001***
**Lower body (P)**	**13.3 (11.1)**	**7.3 (8.2)**	***<0.001***
**Lower body (A)**	**7.2 (7.3)**	**4.3 (5.8)**	***<0.001***
**Lower body (L)**	**8.2 (7.9)**	**3.2 (4.0)**	***<0.001***
**Middle-upper body (F,G)**	**12.9 (15.9)**	**9.1 (11.2)**	***<0.001***
Middle-upper body (F,P)	9.9 (9.7)	7.8 (13.0)	0.055
**Middle-upper body (F,A)**	**10.0 (7.3)**	**6.4 (7.4)**	***<0.001***
**Middle-upper body (F,L)**	**7.9 (6.9)**	**4.6 (7.7)**	***<0.001***
**Fundus (G)**	**19.9 (17.2)**	**14.4 (14.8)**	***<0.001***
**Fundus (P)**	**12.5 (13.4)**	**7.6 (8.6)**	***<0.001***
**Fundus (A)**	**16.7 (15.7)**	**13.7 (15.8)**	***0.040***
**Fundus (L)**	**8.1 (9.6)**	**4.8 (6.8)**	***<0.001***
Fundus (Fornix)	9.3 (12.4)	8.5 (19.2)	0.613
**Middle-upper body (R,P)**	**12.3 (14.2)**	**8.9 (8.4)**	***0.002***
**Middle-upper body (R,A)**	**12.9 (10.8)**	**10.6 (9.9)**	***0.014***
**Middle-upper body (R,L)**	**9.2 (9.5)**	**6.9 (8.0)**	***0.004***
**Angulus (P)**	**6.6 (7.8)**	**5.1 (6.7)**	***0.025***
Angulus (A)	5.0 (6.0)	4.3 (6.7)	0.200
Angulus (L)	9.6 (9.7)	7.9 (12.5)	0.104

## Discussion

Video showing an EGD procedure with Cerebro running simultaneously. The Cerebro system was modified after the study to include 29 sites (epiglottis and vocal cords were not included in this study).Video 1

Use of AI to assist in diagnostic endoscopy has become increasingly popular. In this study, an AI system was built and validated with the aim of improving compliance with a standardized protocol and procedure quality during EGD. ASGE, ESGE, and Yao et al. 7 have established guidelines to ensure quality of EGD. However, these are often not well followed due to lack of supervision and the cumbersomeness of the protocol. Taking photos for documentation is also time-consuming. This system was built to ensure inspection completeness and adequate inspection time and to achieve automatic photo-documentation. In this clinical trial, the AI system Cerebro improved adherence to a standardized protocol during EGD performed by endoscopists-in-training. With use of Cerebro, inspection completeness and number of photos for photo-documentation increased. This is especially helpful at early stages of training when they are not familiar with the 27 sites, and thus, endoscopists in early training phase were chosen as study subjects. The results of this study suggest that the AI system may act as a supervised learning system during EGD for trainees. The gist of this system is to act as a reminder for the 27 sites so the trainees learn the protocol of 27 sites and gradually build it into a routine practice even without AI. With the help of AI, the trainees were able to attain 92.6% completeness of the EGD procedure.

Adequate inspection time during EGD improves gastric cancer detection 16 17 . Park et al showed that an inspection time of more than 3 minutes significantly improved gastric cancer detection in a cohort of 30,506 patients undergoing EGD 18 . This study showed that use of this system increased inspection time at individual sites, especially at the retroflex view of the fundus, mid-upper body, and incisura. These are common sites for missing gastric cancer 19 20 21 . However, the current system measures adequacy of inspection with time only. Quality of inspection was not assessed. The most objective way of assessing this to compare the increase in detection of pathologies. Due to the low incidence of early esophageal and gastric cancer in Hong Kong, there were only three cancers in both arms. The rate of positive pathology detected, therefore, was not different.

The AI system also automatically captures pictures of all 27 sites for photo-documentation. The evaluated algorithm provides automated photo-documentation, which is regarded as a key performance measure for UGI endoscopy by the ESGE 3 . Application of the algorithm led to more extensive image documentation in this study. Standardized photo- and video-documentation systems may serve as a foundation for future development of AI auto-detection of pathology.

There are several limitations of this study. The sequential nature of the clinical trial makes a learning effect of the subjects during the study inevitable. This confounder was accepted in favor of higher generalizability due to a higher number of subjects, as well as the possibility of measuring a subject-specific effect of AI support. A randomized approach was not favored because there would have been a substantial cross-learning effect between the groups. Second, clinical benefit has not been shown in this study due to the low incidence of esophageal and gastric cancers and the relatively small sample size. Third, this AI model was annotated based on images from Olympus-GIF-H290T gastroscopes. Whether this software can be applied to other systems has not been tested. Last but not least, there have been suggestions about an “unlearning” effect by AI and continued dependence on the system once it has been in use, or in other words, reduced quality after AI is discontinued. This study did not address this question.

Because AI is being rapidly deployed in endoscopy, its integration into training programs is controversial. Current applications of AI in endoscopy have largely been focused on computer-aided detection systems for colonic polyps during colonoscopy 22 . Other applications of AI including introduction of real-time instruction, feedback, and competency assessment through AI are becoming promising 23 . Although pathology detection is important, the basics of adequacy of examination during endoscopy also play a pivotal role in decreasing miss rates of cancers. Rodrigues and Keswani proposed a three-stage approach to integration of AI 22 . The first stage is foundational and centers around didactic content to enhance trainee literacy in AI. The second stage introduces AI applications that provide feedback, assess competence, and track quality metrics. AI can also standardize benchmarks across training programs. In the third stage, trainees are exposed to diagnostic and therapeutic features of AI, designed to augment existing skills. These features include computer-aided diagnosis, real-time decision support, and potentially, simulation-based training with AI-generated scenarios. The AI system Cerebro would fit in well in the second stage, where trainees get real-time feedback on their completeness of EGD, their percentage of completeness, and it can be used as a quality indicator for EGD. Future applications of the technology may involve use of the AI system to provide instructions for scope maneuvers to obtain the view of the missing spot.

## Conclusions

In conclusion, completeness of EGD examination and photo-documentation can be improved by AI-assisted software at early stages of training in endoscopy. The AI system can be a supervised learning system to assist trainees in learning.

## Supplementary Material

Supplementary Material Zusatzmaterial
