# Supplementary material for: Artificial intelligence-assisted esophagogastroduodenoscopy improves procedure quality for endoscopists in early stages of training
Source: Endosc Int Open. 2025 Apr 15;13:a25476645. doi: 10.1055/a-2547-6645 (PMC12042994; doi:10.1055/a-2547-6645)
Supplement: Supplementary file 2 — Supplementary Material [file 10-1055-a-2547-6645_25482592.pdf]

Supplementary material 1. Detailed information about all the models and datasets on building Cerebro

Cerebro Multi-AI System

1. Background

Tracking anatomical landmarks in endoscopic videos is a challenging task. Appearance degradation in endoscopic videos is a common problem caused by various factors such as motion blur, video defocus, water splashes, bubbles, froth, and part occlusion. These factors make it extremely difficult for image-based classifiers and detectors to accurately identify and track anatomical landmarks. To overcome appearance deterioration problems, image classifiers typically use temporal information to improve the classification performance on deteriorated frames. One common approach is to operate on a fixed-density and fixed-length temporal window aggregating pixel level features from neighboring frames such as CNN-LSTM [1], 3DCNN [2], 2-stream CNNs [3].

However, these pixel-level strategies have some limitations. First, the dense sampling strategy used in above methods can be computationally inefficient due to the redundancy of temporal information in videos. Second, anatomical landmark appearances, locations may change irregularly over time and therefore additional noise might be also introduced by intuitive adjacent frame selection. The feature representation of the current frame may only be slightly improved at the cost of massive inefficient computations.

2. Motion-adaptive temporal feature aggregation (MA-TFA)

To address these limitations, we have developed a new framework called Motion Adaptive Temporal Feature Aggregation (MA-TFA). This framework is a collaborative spatio-temporal feature aggregation framework consisting of two modules: (1) spatial aggregation module and (2) temporal-relational aggregation module. Spatial features are first extracted using CNN based feature extractors defined in Sec. 2.1. The spatial aggregation module then enhances each feature on the current feature map through aligned features from nearby frames. However, when scene appearance severely deteriorates, spatial features may be inaccurate and adversely affect subsequent feature enhancement. The Temporal Feature Aggregation(TFA) module solves this problem by enhancing image features using proposal features sampled from the long-term historic frames. The framework adapts to changes in appearance and location of anatomical landmarks over time and uses a stride predictor to adaptively sample informative frames from long-term history. The stride predictor determines whether certain frames should be sampled according to the motion speed of the camera. A special image processing module called localized motion estimation is built to estimate the speed of the camera over time from a sequence of images. Sampling from history is performed using this module. The proposal features are regarded as nodes of a specific graph, and each node aggregates information from its neighborhoods through graph operations. The proposals are enhanced by the mined relation features for better decision making, making it a good complement to image-level feature enhancement, especially for severe appearance deterioration. The MA-TFA Framework outputs: (1) one or more anatomical landmarks detected or negative if no landmark detected; (2) One or more quality events: ‘rinsing required’, ‘inflation required’, ‘suction required’.

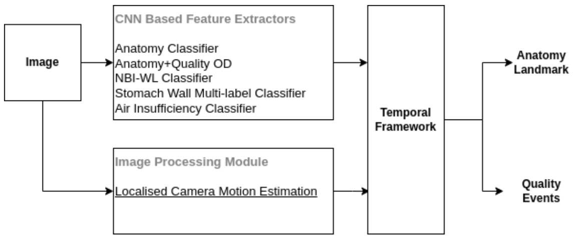

In the above figure, a real-time frame generated from an endoscope is first passed through CNN based feature extractors and Image processing module. These features then become input to the temporal framework which generates the final output. Oftentimes output may contain historic frames.

2.1 CNN-based feature extractors

2.1.1 Anatomy classifier

Classes: Total 33 classes capturing anatomical landmarks *(Hypopharynx area, esophagus tube, stomach body and duodenum part 1 and 2)*. Name of the classes are as follows: mouth, Hypopharynx(epiglottis, vocal Cord), esophagus, esophagogastric_junction, mid_upper_body-a, mid_upper_body-g, mid_upper_body-l, mid_upper_body-p, pyloric_channel, antrum-a, antrum-g, antrum-l, antrum-p, duodenum1, duodenum2, fundus_cardia-a, fundus_cardia-g, fundus_cardia-l, fundus_cardia-p, fornix, incisura-a, incisura-l, incisura-p, lower_body-a, lower_body-g, lower_body-l, lower_body-p, mid_upper_body_retroflex-a, mid_upper_body_retroflex-l, mid_upper_body_retroflex-p, negative, in_vitro.

Datasets:

	Training	Validation	Total
**Frames**	422836	1012128	1434964
**Videos**	250	302	552

Data Preprocessing: Image pixel values are normalized (div by 255). All images are resized to 224x224x3 size. Two sets of main augmentations/transformations are applied: (1) class-wise augmentation: applied to some classes; (2) common augmentation: applied to all classes. class-wise augmentation: (1) Flipping: Applied at a probability of 80%, flips frames vertically/horizontally/both.
(2) Rotation: Applied at probability of 30%, rotates images in range (-30,30) degrees, and extends the remaining border with black pixels. The individual probabilities of the flip and rotate augmentations are normalized to 1 and act as weights.

Common augmentation: This augmentation is applied to all classes. It consists of one of: *GridDropout*; *CutOut*; *ColorJitter*; *Rotate; ShiftScaleRotate*;

Training algorithm: Core architecture used is an EfficientNet-B7 [4] with pre-trained weights trained on ImageNet21k dataset. Pre-trained EffNet-B7 is used as is for the convolutional backbone/feature extractor followed by Softmax activation. The output from the EffNet base is the prediction output pred. For the MLP we customize a sequential module with: Dropout layer (0.2 dropout), Linear transform layer, ReLU activation layer, Linear transform layer.

The loss while training, is a weighted sum of three losses. (1) Supervised Contrastive Loss [5]; (2) Binary Cross Entropy Loss; (3) Asymmetric Generalized Cross Entropy Loss; The net loss (while training) is defined as:

$$total\_loss = (1.5 * ce\_loss) + sup\_con\_loss + agce\_loss$$

For optimizers we used a Ranger optimizer [6]. The learning rate(LR) is set 8 times during the run (including the initial LR), spaced equally every 15 epochs. The scheduler is a list of sequential CosineAnnealingLR schedulers; one for every updated LR. The model is trained for 120 epochs, with batch size of 8.

Results:

Macro F1-score	Top-1 accuracy	Top-2 acuracy
0.651	0.64	0.81

2.1.2 Anatomy object detector

Classes: pyloric valve, device, incisura, antrum muscle, esophagus_cardia_junction, esophagus, incisura_arc_lower_body, incisura_arc_middle_upper_body', d1_bulb, d2_center, froth, food_residue, water jet, filled water area

Training algorithm: We start with Yolov5m [7] (medium sized) pre-trained model and train it for 100 epochs with no frozen layers on our dataset on a batch size of 8 and image size 416x416. For inference Confidence threshold of 0.25 and Iou Threshold of 0.45 was used. Training IOU threshold used is 0.4.

Results:

Precision	Recall	mAP50
0.651	0.572	0.572

2.1.3 Stomach Wall Multi-label Classifier

The purpose of this model is to classify stomach wall(s) visible in the image. Multiple walls can be present in the same image indicating multiple walls being inspected simultaneously. There are 2 models trained separately for antegrade and retroflex respectively.

Classes: Antegrade Model: A, G, L, P, Antrum, Lower Body(LB), Mid Upper Body(MUB).
Retroflex Model: A, G, L, P, Mid Upper Body Retroflex(MUBR), Fundus Cardia(FC).

Datasets:

	Training	Validation	Total
**Frames**	38192	16408	54600
**Videos**	106	40	146

Training algorithm: Both models are trained with the same method. Architecture used was a customized ResNet34[8]. The backbone is the convolutional layers of the pretrained ResNet34 trained on the ImageNet1K-v1 dataset. For the feature extractor we use the ResNet where we only employ the convolutional layers with pretrained weights, and discard the last fully connected linear transformation layer. For the classifier we construct a custom fully connected layer, with no pretrained weights. Loss function used is Asymmetric Loss Optimized, for multi-label classification with sum reduction. The output vector of the retroflex model looks like ["A", "G", "L", "P", "MUBR", "FC"]. Where the first 4 values predict the multi-label probability of which sites are positive; and the last 2 predict a binary class probability of which area is positive. Similarly for antegrade, [“A”, “G”, “L”, “P”, “MUB”, “AN”, “LB”]. Therefore, for the prediction y_hat , we applied sigmoid activation on y_hat[:4], and softmax activation on the remaining y_hat[4:]. Model is trained for 50 epochs.

Results: Metrics below are averaged across the entire validation epoch. Accuracy is calculated only for the multi-label ALGP classes (i.e. excluding the predictions for area classes FC, MUBR, Antrum, LB, MUB)

	Antegrade model	Retroflex model
**Accuracy**	0.849	0.8204
**Precision**	0.8516	0.8126
**Recall**	0.8411	0.8274
**F1-Score**	0.8442	0.81

2.1.4 NBI-WL classifier:

Classes: NBI (Narrow-band Imaging), WL(white-light)

Datasets:

	Training	Validation	Total
**Frames**	40542	10136	50678
**Videos**	60	45	105

Training algorithm:
The primary architecture is a MobileNetV3_small [9] with pre-trained weights. Followed by a ReLU activation and a linear transformation layer which outputs a prediction logit vector of size 2 (for 2 classes).
Results: Accuracy: 0.987

2.1.3 Air insufficiency classifier

Classes: Sufficient-Air, Insufficient-Air

Datasets:

	Training	Validation	Total
**Frames**	14343	10136	24479
**Videos**	35	20	55

Training algorithm: EfficientnetB0 [4] is used with default hyperparameters and pre-trained weights. Model is trained for 30 epochs.

Results:

Precision	Recall	Accuracy	F1-Score
96.5	95.26	95.83	95.88

3. Performance of MA-TFA Framework

Performance of MA-TFA framework is measured based on its ability to correctly identify false positives(FPs) and true positives(TPs) from the output of image based models. To test the performance we created a dataset of FPs and TPs from inferences of CNN models from Section 2.1 on fully labeled videos.

Test dataset:
Total of 44 videos used for benchmarking the performance of the framework.

	TP	FP	Total
**Frames**	69292	17332	86624

Test results:
MA-TFA Framework is able to correctly classify anatomy-classifier's inferences as FP or TP with very high confidence.

	Precision	Recall	F1-Score
**FP**	0.89	0.92	0.86
**TP**	0.81	0.85	0.83

Improvement in overall accuracy of Landmark Detection using MA-TFA framework.

	Top-1 accuracy (landmark detection)
**Anatomy classifier**	0.64
**MA-TFA**	0.88

4. References

1. *Sainath TN, Vinyals O, Senior A* et al. Convolutional, long short-term memory, fully connected deep neural networks. 2015 IEEE International Conference on Acoustics, Speech and Signal Processing (ICASSP), South Brisbane, QLD, Australia, 2015: 4580-4584, doi: 10.1109/ICASSP.2015.7178838. (2015)

2 Tran D, Bourdev D, Fergus R et al. Learning spatiotemporal features with 3D convolutional networks. IEEE transactions on pattern analysis and machine intelligence. 2017. arXiv:1412.0767

3 Simonyan K, Zisserman A. Two-stream convolutional networks for action recognition in videos. Advances in neural information processing systems. 2014. arXiv:1406.2199

4 Mingxing T, Quoc V. EfficientNet: Rethinking model scaling for convolutional neural networks. arXiv:1905.11946

5 Khosla P, Teterwak P, Wang C et al. Supervised contrastive learning. Advances in neural information processing systems. 2020. arXiv:2004.11362

6 Liu L, Jiang H, He P et al. On the variance of the adaptive learning rate and beyond. 2019. arXiv:1908.03265

7 Redmon J, Santosh D, Girshick et al. You only look once: Unified, real-time object detection. Proceedings of the IEEE conference on computer vision and pattern recognition, 2015. arXiv:1506.02640

8 He K, Zhang X, Ren S et al. Deep residual learning for image recognition." Proceedings of the IEEE conference on computer vision and pattern recognition. 2016. arXiv:1512.03385

9 Howard AG, Zhu M, Chen B et al. MobileNets: Efficient convolutional neural networks for mobile vision applications. 2017. arXiv:1704.04861

Supplementary material 2 Time spent on each location during EGD for both groups.

Inspection time	AI-assisted (n = 233) Mean time in seconds (SD)	Control (n = 233) Mean time in seconds (SDd)	*P* value
Overall	776.7 (341.8)	745.4 (267.9)	0.272
Esophagus	22.1 (26.3)	20.8 (17.8)	0.536
Squamocolumnar junction	17.5 (23.4)	16.3 (19.1)	0.520
Antrum (G)	39.2 (27.8)	35.1 (23.1)	0.079
Antrum (P)	29.4 (21.5)	25.8 (20.3)	0.065
Antrum (A)	27.1 (24.5)	22.8 (22.6)	0.051
Antrum (L)	31.4 (25.5)	25.5 (23.8)	0.011
Duodenal bulb	14.7 (15.2)	13.7 (12.1)	0.417
Duodenal descending	31.8 (34.4)	35.9 (33.8)	0.196
Lower body (G)	13.1 (10.0)	7.9 (9.1)	< 0.001
Lower body (P)	13.3 (11.1)	7.3 (8.2)	< 0.001
Lower body (A)	7.2 (7.3)	4.3 (5.8)	< 0.001
Lower body (L)	8.2 (7.9)	3.2 (4.0)	< 0.001
Middle-upper body (F,G)	12.9 (15.9)	9.1 (11.2)	< 0.001
Middle-upper body (F,P)	9.9 (9.7)	7.8 (13.0)	0.055
Middle-upper body (F,A)	10.0 (7.3)	6.4 (7.4)	< 0.001
Middle-upper body (F,L)	7.9 (6.9)	4.6 (7.7)	< 0.001
Fundus (G)	19.9 (17.2)	14.4 (14.8)	< 0.001
Fundus (P)	12.5 (13.4)	7.6 (8.6)	< 0.001
Fundus (A)	16.7 (15.7)	13.7 (15.8)	0.040
Fundus (L)	8.1 (9.6)	4.8 (6.8)	< 0.001
Fundus (fornix)	9.3 (12.4)	8.5 (19.2)	0.613
Middle-upper body (R,P)	12.3 (14.2)	8.9 (8.4)	0.002
Middle-upper body (R,A)	12.9 (10.8)	10.6 (9.9)	0.014
Middle-upper body (R,L)	9.2 (9.5)	6.9 (8.0)	0.004
Angulus (P)	6.6 (7.8)	5.1 (6.7)	0.025
Angulus (A)	5.0 (6.0)	4.3 (6.7)	0.200
Angulus (L)	9.6 (9.7)	7.9 (12.5)	0.104

Supplementary material 3 Table showing malignant pathologies in the two groups.

**Malignant lesions**	**Control group (n = 233)**	**AI-assisted group (n = 233)**
Esophageal cancer		
Gastric cancer	3	1
Duodenal cancer	0	2
Subepithelial lesions	1	1
	4	1

Endosc Int Open | DOI 10.1055/a-2547-6645 | © The Author(s).
